# Gaze Control of a Robotic Head for Realistic Interaction With Humans

**DOI:** 10.3389/fnbot.2020.00034

**Published:** 2020-06-17

**Authors:** Jaime Duque-Domingo, Jaime Gómez-García-Bermejo, Eduardo Zalama

**Affiliations:** ITAP-DISA, University of Valladolid, Valladolid, Spain

**Keywords:** gaze control, gaze engagement, HRI, humanoid robot, robotic head, ROS, competitive network, computer vision

## Abstract

When there is an interaction between a robot and a person, gaze control is very important for face-to-face communication. However, when a robot interacts with several people, neurorobotics plays an important role to determine the person to look at and those to pay attention to among the others. There are several factors which can influence the decision: who is speaking, who he/she is speaking to, where people are looking, if the user wants to attract attention, etc. This article presents a novel method to decide who to pay attention to when a robot interacts with several people. The proposed method is based on a competitive network that receives different stimuli (look, speak, pose, hoard conversation, habituation, etc.) that compete with each other to decide who to pay attention to. The dynamic nature of this neural network allows a smooth transition in the focus of attention to a significant change in stimuli. A conversation is created between different participants, replicating human behavior in the robot. The method deals with the problem of several interlocutors appearing and disappearing from the visual field of the robot. A robotic head has been designed and built and a virtual agent projected on the robot's face display has been integrated with the gaze control. Different experiments have been carried out with that robotic head integrated into a ROS architecture model. The work presents the analysis of the method, how the system has been integrated with the robotic head and the experiments and results obtained.

## 1. Introduction

The gaze control of a robotic head represents an important field of research in robotics, since it promotes higher evaluations of a robot's comprehension and naturalness (Kousidis and Schlangen, 2015) in human-robot interaction. This gaze engagement represents a key factor in interaction because humans feel more comfortable if robots behave like a person. Some robots look and track people, but are not able to change between several interlocutors during a conversation. If someone disappears from the field of view, the robot listens to a sound and turns its head, looking for someone to follow. This behavior is not natural in a conversation of several people.

The method proposed in this article responds to the problem of several individuals interacting with a robotic head. It replicates human behavior using a competitive neural network which receives stimuli from each person who interacts with the robot. Different factors are taken into account: look, who speaks, pose, hoard conversation, habituation, etc. These factors produce stimuli which create a dynamic conversation, independently of whether several people appear and disappear from the visual field. Different considerations have been taken into account:

- There may be several people in front of the robot.
- One or several people could speak at the same time.
- People could enter and exit the visual field of the robot.
- People could appear and disappear from the scene due to occlusions or false detections.
- People may be looking at the robot or elsewhere.
- People must be distinguishable from each other to facilitate their monitoring.
- People could request the robot's attention in several ways (entering their visual field, talking, moving in front of the robot).
- The robot should give more attention to new stimuli (e.g., a person starts talking, while another one has been talking for a certain time).
- People can be in different image planes (closer or farther from the robot).
- The transition of the change between two people should be smooth and weighted, avoiding jerky movements.

The proposed method is based on a competitive network that accurately combines a set of identification techniques, facial monitoring, and behavioral rules to achieve the most natural interaction. The system deals with the presence of different stimuli and allows a stable determination of the focus of attention that must be followed with the robot's eyes. At the same time, the system has principles of adaptation and stability. The robot must be able to respond quickly to new stimuli, but at the same time the response must be smooth and stable, avoiding erratic behaviors.

The robot's gaze control not only falls on the movement of the head. A projected virtual face, hereinafter referred to as the agent, has been created to move the eyes and show expressions based on such factors as the presence of people. As an example, if there is no one in front of the robot, the agent will show a sad expression and begin an exploratory movement. In Ishi et al. (2010), an experiment with two different robots showed a more natural behavior of one of them just because the ability to move the lips.

The present paper is structured as follows: section 2 explores the state-of-art of the technologies considered in this paper. Section 3 shows how the method works, exploring the different steps: face recognition, people pose, speaking detection, competitive network, etc. Section 4 explains how this method has been integrated with a robotic head developed for this purpose. In section 5, the different experiments and results obtained with the robotic head are reported. An overall discussion on the obtained results is stated. Finally, section 6 notes the advantages and limitations of the presented system and suggests future developments.

## 2. Overview of Related Work

Gaze control has been an important field of research over the last few years since it contributes to the improvement of communication with people. As stated by Kousidis and Schlangen (2015), when a robot is a listener in a multi-party conversation and tracks the conversation with its gaze, it promotes higher evaluations of that robot's comprehension and naturalness than a robot performing random gazing between speakers. Moreover, Garau et al. (2001) proposed that virtual agents which use turn taking gaze during conversations are evaluated as more natural and pleasant than agents that use random gaze or none gaze control in their communication. What is more, their conversation is rated as more engaging. Boucher et al. (2012) studied the gaze effects of Human-human interaction in a cooperation experiment and implemented a heuristic capability to generate such gaze cues by a humanoid robot. However, that work was mainly focused on the interaction with just one user. In addition, as studied by Andrist et al. (2015), the gaze behavior more effectively motivates users to repeatedly engage in therapeutic tasks.

Neurorobotics plays an important role in Human–Robot Interaction (HRI), a discipline that allows improving robots which can communicate and respond to ongoing human communications and behavior (Kiesler and Hinds, 2004). It also plays an essential role in assistive and rehabilitation robotics (Beckerle et al., 2017). Admoni and Scassellati (2017) have recently presented a survey of the state of the art in social eye gaze for HRI. The authors distinguish between three different approaches to the problem: Human-focused, centered on understanding the characteristics of human behavior during interactions with robots; Design-focused, which studies how the design of a robot impacts on interactions with humans; and Technology-focused, with the aim of researching how to build computational tools to guide the robot's gaze in human interaction. According to the authors, the main challenges of conversation are managing attention and turn-taking between partners, selecting the correct gaze for the conversational content, and adopting the right conversational roles. In addition, Thrun (2004) indicated that the shape of the robot, specifically humanoid features, influences people's behavior toward the machine and their expectations about its capabilities.

Regarding gaze control, an outstanding work was published by Zaraki et al. (2014), where the authors created a system to guide a robot's gaze at multiple humans who were interacting with the robot. The attention mechanism used features which had been proven to guide human attention. The authors relied on the use of a Kinect sensor to track people and obtain sound direction. This system considers the maximum of the sum of different elements: social features, proxemics values, orientation, and a memory component. However, the stimuli considered were limited and the maximum value could change abruptly and could also lead to erratic changes in the focus of attention. Our proposal does not need a 3D sensor and is able to work with common RGB cameras, while a competitive neural network provides soft transitions between different focuses of attention. Another remarkable work was published by Alonso-Mart́ın et al. (2012), who used 8 different microphones in a social robot, named Maggie, to determine which direction to look. Once the orientation of the robot with respect to the user was obtained, an infrared laser returned the distance with respect to him/her to make the robot move forward/backward. That work was able to guide the robot in the direction of a speaker, but did not consider face stimuli or a competitive behavior. A robot equipped with two laser range-finders was also used by Schulz et al. (2003) to probabilistically track the position of people with a mobile robot, although it did not have a cognitive behavior. Saldien et al. (2014) presented a robot focus on Robot Assisted Therapy (RAT) which was able to perceive different stimuli (visual, auditory, and tactile) and track a certain colored object, a face or a directional voice. However, that work did not solve the problem of who to pay attention. Vega et al. (2013) proposed a dynamic visual memory to store the 3D processed information from a moving camera on board the robot. The attention system chose where to look, according to the principles of reobserving objects in the visual memory and the need to explore new areas. The visual memory was a collection of relevant task-oriented objects and 3D segments. However, that work was mainly focused on creating a visual memory about objects and reobserving them according to the basis of keeping the memory updated. More recently, Viciana-Abad et al. (2014) have demonstrated the benefits of fusing sensory information with Bayes inference. The authors localize a person with a robotic head by simultaneously processing visual and audio data. The authors mainly focus on tracking a particular person instead of a conversation between multiple participants.

Visual information represents an important aspect of HRI, increasing the robot's awareness. The robots must trade off features which affect the utility of the visual information (Gergle et al., 2013), such as the robot's field of view (FOV), alignment of perspective, degree of spatial resolution, frame rate or synchronization with a voice stream. The Visual Focus of Attention (VFOA) represents who or what people are looking at. Massé (2018) presents a VFOA model based on a Bayesian network to infer the relation between head poses and object locations. That work exploits the concept of correlation between eye gaze and head movements instead of using face landmarks. The author uses a convolutional neural network to predict object locations and a reinforcement learning method for robotic gaze control. The robot autonomously learns a strategy for moving its head using audio and visual observations. The author mainly focuses on the relation between head poses and objects and not the conversations between different participants. In Ghiţă et al. (2018), the authors track people in a robot assistive care scenario by using an *Oriented FAST and Rotated BRIEF* detector (ORB) and comparing characteristics between frames. According to their study, NAOqi functions, which are SO/API for Pepper, NAO and Romeo robots, are improved, covering more range, situations of occlusions and more orientations of a person/face. Shiomi et al. (2004) develop a face to face tracking of people, generating hypotheses about people position by using peripheral vision. Even though a face may not be present in the foveal vision of the robot when it is gazing at another object, the robot keeps plausible hypotheses about the location of the human faces. However, in Csapo et al. (2012), the authors point out some problems with Nao platform about non-verbal human-robot interaction, where some capabilities of detecting faces or tracking people interfere with other modules that send commands to the same motor, producing senseless movements due to conflicting signals.

Regarding the identification of people, the feature-based systems play an important role in both human and robotics perception (Potapova et al., 2017). Different techniques have been developed for face detection and recognition during the last years. As stated by Zafeiriou et al. (2015), robust feature extraction methodologies have been used for face detection, such as *Scale Invariant Feature Transform* (SIFT) features (Lowe et al., 1999; Geng and Jiang, 2009; Lenc and Král, 2015), *Histograms of oriented Gradients* (HoGs) (Dalal and Triggs, 2005), *Local Binary Patterns* (LBPs) (Ahonen et al., 2006), or *Haar cascade classifiers* (Viola and Jones, 2004). Among the most advanced techniques, Haar classifiers, HoG detector or Deep Learning based solutions are widely used, as they are implemented in OpenCV or DLIB libraries. Haar classifiers detect faces at different scales but do not work with non-frontal faces and occlusions and return a large number of false predictions. The HoG feature descriptor is fast but does not detect small faces (less than 80 ×80 pixels). It can work with some minor occlusions or non-frontal faces, but returns a bounding box that often excludes part of the forehead/chin. The DLIB library (King, 2009) implements a CNN face detector using a Maximum-Margin Object Detector (King, 2015), which works for different face orientations and occlusions. However, it does not return a precise bounding box real-time. OpenCV offers a DNN Face Detector, based on a Single-Shot-Multibox detector (Liu et al., 2016), which is very accurate, works with different face orientations, scales and occlusions, and runs in real-time on CPU. Another important aspect of face recognition is the extraction of the face's features. They are mainly obtained using an *Active Appearance Model* (Cootes et al., 2001; Milborrow and Nicolls, 2008) or DLIB-68 model (Kazemi and Sullivan, 2014), which makes a face alignment with an ensemble of regression trees before obtaining the corresponding landmarks. Finally, the face recognition is mainly based on Deep Residual Learning algorithms, which are very accurate. DLIB implements a ResNet network with 29 convolution layers and uses a pre-trained model which takes the 68 face landmarks obtained from an image (Kazemi and Sullivan, 2014).

As in the case of facial recognition, human body recognition relies on the use of *Haar filters* (Viola et al., 2001), HoG (Dalal and Triggs, 2005), or *Deep Convolution Neural Networks* (DCNN), such as the *Faster RCNN Inception V2 COCO Model* (Ren et al., 2015). All of them are available in OpenCV, working simultaneously with TensorFlow. Systems based on DCNN offer better results, detecting more people with a lower number of false positives.

Lip activity detection has also been studied in different works. Bendris et al. (2010) proposed a method to detect lip motion by measuring the degree of disorder of pixel directions around the lip using the optical flow technique (Saenko et al., 2005). A rectangle around the lips was enlarged to be aligned with a previous one by taking the region that minimized the mean squared difference (MSD). Siatras et al. (2008) considered the increased average value and standard deviation of the number of pixels with the lowest intensities of the mouth region to detect visual speech. They created a statistical algorithm that used two detectors based on noise to characterize visual speech and silence in video sequences.

Neural networks have played a major role in the interaction of robots with people. An outstanding work was presented by Bicho et al. (2010), who presented a control architecture for human-robot collaboration which was formalized by a coupled system of dynamic neural fields representing a distributed network of neural populations that encode in their activation patterns goals, actions, and shared task knowledge. This approach was valid for inferring the response to user stimuli but was only valid for the collaboration with one user. In our paper, a competitive network is used to create a dynamic behavior in the interaction between several participants. A competitive network consists of a layer that is able to react to different stimuli and decide a winner. This network has a progressive behavior which does not switch sharply between consecutive winners. A habituation layer avoids a participant from being the winner for a long period of time when a new, different stimulus arrives. For example, imagine that two people are talking and one of them is monopolizing the conversation. The robot would gaze at the person who monopolizes the conversation but, if the other person says something, a more natural behavior is to gaze at the new interlocutor.

## 3. Analysis of the System

**Figure 1:**
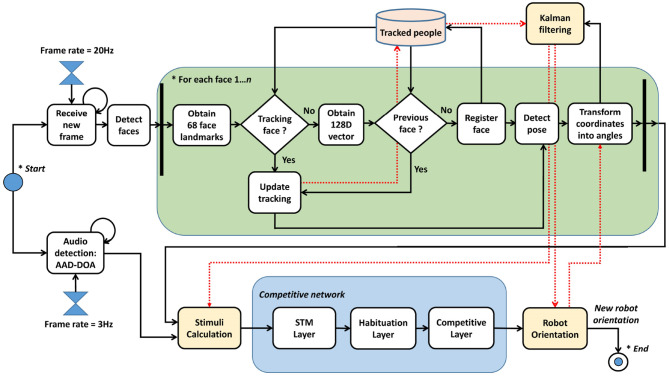
Steps in the gaze control process.

The method proposed in this section analyzes how the gaze control of a robotic head works. Figure 1 shows the different steps needed to obtain the orientation angles of the robotic head.

There is a specific frame rate that determines how many images are received per second. Whenever a frame is received, there is a process of people detection and face landmarks are obtained using the DLIB 68 model (Kazemi and Sullivan, 2014). There is a temporal table which memorizes the people who have interacted with the robot. If a person is new, a 128D vector is obtained by techniques related to facial recognition, as explained later. This vector allows a concrete person to be identified. Once a person has been identified, the method uses correlation tracking (Danelljan et al., 2014) to follow the person during consecutive frames. If a person disappears from the scene for a while, the method is able to keep their 128D vector in its memory to make a subsequent re-identification. Different stimuli, such as visual speaking detection and pose estimation, are evaluated for each person, and they represent the entry of a competitive network composed of three different layers:

- *Short Time Memory layer (STM)*, which extends the duration of the concrete stimuli. As an example, if a person says a short sentence, the system might not properly consider the stimulus. This layer extends the duration of the stimuli to have more value in the entry of the habituation layer.
- *Habituation layer*, which penalizes persistent stimuli against new stimuli, e.g., it prevents someone from hoarding the conversation. When a person is speaking continuously, if someone says something, it makes it easier to pay attention to the new interlocutor.
- *Competitive layer*, which is the final step of the network. The input to the competitive network includes stimuli for each person in the memory, and decides who is the winner. This layer is responsible for following a dynamic behavior, without abrupt jumps.

Kalman filters are used to process the position of people over time. These filters have been previously used on robotic heads, such as Milighetti et al. (2011), who predicts the next state of the moving target. In our work, 2D face positions obtained during tracking are transformed into concrete angles of the robotic head (ψ_*c*_ and θ_*c*_). These angles are integrated in different Kalman filters, one for each person in memory, giving the position of the person independently of being in the robot's FOV. After a winner is provided by the competitive network, the new orientation of the robotic head is that returned by its Kalman filter. Then, the Kalman filters are updated with the last positions obtained.

The rest of this section is composed of different subsections: section 3.1 explains the different stimuli considered and how they are calculated. Section 3.2 presents how the competitive network works. Section 3.3 shows how the face coordinates of a person obtained from an image are transformed into the angles of the robot. Finally, section 3.4 presents how the Kalman filter is used.

### 3.1. Entry Stimuli of Competitive Network

Each person, *k*, produces a set of stimuli, *x*. This set of stimuli are introduced into the competitive network and defined *I*_*kx*_. Several stimuli that consider how people react in a conversation have been used. The stimuli that can be present or absent are coded as binary values and are balanced in importance by a weight, *w*_*kx*_:

- *I*_*k*1_ is the stimulus associated to a person *k* who is situated in the robot's field of view (FOV). People situated in front of the robot are candidates to be interacting with the robot. *w*_*k*1_ is the corresponding weight associated to that stimulus.
- *I*_*k*2_ is the stimulus associated to a person *k* who is considered to be speaking. Lip movement detection is performed, based on mouth landmarks. Moreover, in order for a person to be considered as a speaker, incoming audio has to be detected in its direction.
- *I*_*k*3_ is the stimulus associated to a person *k* who is gazing directly at the robot. The pose is an important stimulus which indicates that a person is visually interacting with the robot. This stimulus represents the *mutual gaze*, a kind of *shared looking* which is related to the increase of the engagement in the interaction (Sidner et al., 2004).
- *I*_*k*4_ is the stimulus associated to a person *k* who is continuously moving. In a conversation with several people, an individual tends to look at another restless person. This stimulus requires the individual to be situated in the FOV of the robot. If the sum of differences of a person's position between several frames is over a concrete threshold, the person is considered to be restless.
- *I*_*k*5_ is the stimulus associated to a person *k* who is not situated in the robot's FOV, but for whom audio has been detected. When a person is interacting with a group of people and someone is speaking at their left/right side, this individual tends to turn the head in that direction looking for the person.
- *I*_*k*6_ is the stimulus associated to a person *k* who is not situated in the robot's FOV, but who is the VFOA of another group of people. When two or more people in the FOV are gazing in the same direction, a stimulus is given to people in that direction. In this situation, this stimulus considers the Visual Focus of Attention (VFOA).
- *I*_*k*7_ is the stimulus associated to a person *k* who is situated at a certain distance, following a proxemic approach as in other works (Alonso-Mart́ın et al., 2012; Zaraki et al., 2014). This stimulus is multiplied by an adjustment factor which depends on the distance between the person and the robot.

Next, the calculus of each stimulus is explained.

#### 3.1.1. Stimulus *I*_*k*1_: Person in the FOV of the Robot

The robot detects faces in frames and persons are, first of all, recognized and labeled. When the robot turns its head, if a new face appears on the FOV, the robot can discern whether this person was previously recognized. When a face is in the FOV during a period of time, re-identification is not required since the face is followed by tracking.

There are different techniques for face recognition, but those based on Deep Residual Learning are very accurate. The library DLIB implements a ResNet network with 29 convolution layers. This model is similar to the ResNet-34 network (He et al., 2016) with a few layers removed and the number of filters per layer reduced by half. This library uses a pre-trained model and takes the 68 face landmarks obtained from an image (Kazemi and Sullivan, 2014), aligns the face and maps it to a 128 dimensional vector space where images of the same person are close in terms of distance. Once the 128D vector has been obtained, the similarity of two faces is calculated, checking if their Euclidean distance is small enough. Using a threshold of 0.6, the DLIB model obtains an accuracy of 99.38% on the standard LFW face recognition benchmark (Huang et al., 2008). This procedure requires the use of GPU, due to the fact that obtaining the 128D vector from a face takes more than 0.4 s with CPU. With an intel i9-9900K and a GPU GeForce RTX 2080 Ti, the 128D vector is obtained in less than 0.05 s.

When a person is identified, a tracker is used to follow the person in the FOV. It increases the speed of the system, as tracking is faster than face recognition. The DLIB library allows a correlation tracker based on Danelljan et al. (2014) to be used. This method considers the approach of Bolme et al. (2010) and makes use of learning discriminative correlation filters based on a scale pyramid representation. The authors use separate filters for tracking, in real-time, objects that change in both translation and scaling.

#### 3.1.2. Stimulus *I*_*k*2_: Person Speaking in the FOV of the Robot

As stated in the overview of related work, there are different works which have studied lip activity to discern whether a person is speaking or not. Speaking detection is important in a conversation because, when a person is looking at the robot but is in silence, it is likely that another person who is speaking receives the robot's attention.

**Figure 2:**
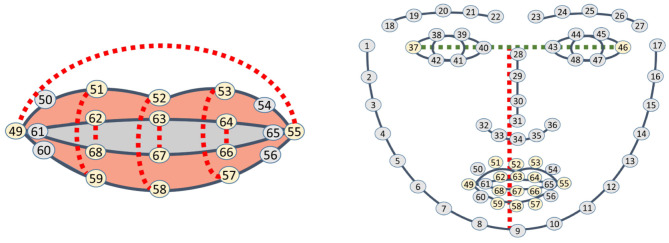
Visual speaking detection. **(A)** Points used for lip activity detection. **(B)** Points to normalize distance.

Instead of implementing a complete analysis of images as in Bendris et al. (2010) or Siatras et al. (2008), and due to the fact that DLIB 68 returns characteristic points of the mouth (points 49–68), the movement between lips is calculated in consecutive frames using these points. The sum of distances between a group of points (62 and 68, 63 and 67, 64 and 66, 49 and 55, 51 and 59, 52 and 58, 53 and 57) (see Figure 2A) is divided by the distance between the middle point of the eyes, points 37 and 46, and the end of the chin, point 9 (see Figure 2B). This division is done to normalize the distance, regardless of whether the person is close or far away.

Let *d*_*k*_ be the result of the calculus of the separation of lips in frame *k*, as shown in Equation (1).

(1) $$\begin{array}{l} d_{k}=\frac{\left|\vec{P_{62},P_{68}}\right|+\left|\vec{P_{63},P_{67}}\right|+\left|\vec{P_{64},P_{66}}\right|}{\left|\vec{P_{m}(P_{37},P_{46}),P_{9}}\right|}\\ \;+\frac{\left|\vec{P_{49},P_{55}}\right|+\left|\vec{P_{51},P_{59}}\right|}{\left|\vec{P_{m}(P_{37},P_{46}),P_{9}}\right|}\\ \;+\frac{\left|\vec{P_{52},P_{58}}\right|+\left|\vec{P_{53},P_{57}}\right|}{\left|\vec{P_{m}(P_{37},P_{46}),P_{9}}\right|} \end{array}$$

*d*_*k*_ is added during a few consecutive frames, say 5 frames. If the result is over a threshold τ_*S*_, that is $\overset{5}{\underset{k=1}{\sum }}d_{k}\geq \tau_{S}$, the person is visually considered to be speaking.

After the visual speaking confirmation, it is necessary to evaluate if there is audio in the direction of that person. A person can move his/her lips, i.e., breathing, but in silence. An Audio Activity Detection (AAD), combined with a Direction of Arrival system (DOA) (Griffin et al., 2012), is used to detect the zone where audio is originated (left, central, right). When the person is visually speaking in the robot's FOV and audio is detected in the direction of the robot's gaze (80°), that person is considered to be speaking.

#### 3.1.3. Stimulus *I*_*k*3_: Person Gazing Directly at the Robot

The determination of people pose is an important stimulus because, when a person is looking at the robot, there is a greater interaction between both participants: person and robot.

Instead of developing a complete analysis of image, such as Ba and Odobez (2008), where the authors study how to link head position with the visual focus of attention, modeling the pose observations with a Gaussian Mixture Model (GMM) or a Hidden Markov Model (HMM), the use of known face landmarks is exploited. A Perspective-n-Points algorithm (PnP) associates 2D points of the DLIB 68 model (Kazemi and Sullivan, 2014) with 3D points in a respective model. Using a standard 3D model of a head, with some characteristic points such as nose tip, chin, left eye left corner, right eye right corner, mouth left corner or mouth right corner, it is possible to calculate the respective pose between the DLIB points and the 3D model. PnP is implemented in different ways, but the solution DLT + Levenberg-Marquardt Optimization has been chosen. A *Direct Linear Transformation* (DLT) algorithm allows *L*, the projection matrix of the camera, to be calculated. In the formula *LX*_*i*_ = *u*_*i*_, 2D coordinates *u*_*i*_ are related to a 3D point *X*_*i*_. To obtain *L*, the six different points selected (nose tip, chin, etc.) and the 3D position of these points in the model are introduced in the algorithm. The Levenberg-Marquardt Optimization finds a pose that minimizes the re-projection error, which is the sum of the squared distances between the observed image points and the projection.

**Figure 3:**
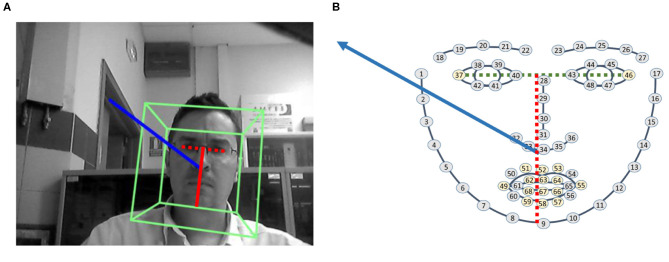
Pose module and distance between eyes and chin. **(A)** Pose of a person. **(B)** Pose vector and DLIB landmarks.

When the pose has been determined, and considering the nose tip as the origin of coordinates, a 3D vector from the origin is projected onto the 2D image: say *x*_1_(0, 0, 0)−*x*_2_(0, 0, 500) projected as *u*_1_(*x*_1_, *y*_1_)−*u*_2_(*x*_2_, *y*_2_). The module of this vector, $\left|\vec{u_{1},u_{2}}\right|$, which is represented in blue in Figure 3, is divided by the distance between the middle point of the eyes, points 37 and 46, and the end of the chin, point 9 (red vector). This division normalizes the module, making it independent of the distance to the face. If the result is below a given threshold, τ_*M*_ <5, the person is considered to be looking at the robot and their stimulus is increased in the entrance of the competitive network.

#### 3.1.4. Stimulus *I*_*k*4_: Person Continuously Moving

In a conversation with several people, a person tends to look at another restless one. The angles associated to a person, ψ_*c*_ and θ_*c*_, are stored during 25 consecutive frames, as this number of frames has produced the most accurate results. The difference between each pair of consecutives angles is calculated and accumulated. These differences are computed using the Euclidean distance. If the accumulated distance is over a concrete threshold, τ_*M*_, the person is considered to be a restless person.

#### 3.1.5. Stimulus *I*_*k*5_: Person Not in the Robot's FOV but With Audio

In a conversation with several people, a person has to turn the head left/right when other persons are speaking in that direction. As explained before, the proposed method uses Kalman filters to keep the last estimated position of each person in memory. The DOA system (Griffin et al., 2012) indicates the direction of audio: left, central, right. When nobody is speaking in the robot's FOV, all people situated to the left/right side of the robot, according to the person's angles and robot pose, receive stimulus if audio has been detected in their zone.

In addition, there are two fictional persons who are situated in the left/right zone of the robot, respectively. When a new participant begins speaking, who has not been previously detected by the robot, the corresponding fictional person receives stimulus whenever audio is detected in their zone and nobody is speaking in the robot's FOV. This is used to integrate that person in the competition and be able to get the robot's attention.

The DOA system is also used when the robot has not previously detected any person. When a sound arrives from a concrete direction, the robot will begin an exploratory movement in that zone to search for people.

#### 3.1.6. Stimulus *I*_*k*6_: Person in the VFOA of Other People

**Figure 4:**
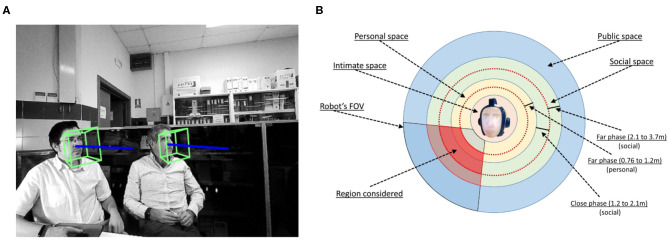
VFOA and proxemic. **(A)** VFOA of two people. **(B)** Considering region in the proxemic approach.

As assumed by other authors (Massé, 2018), it is important to consider the *Visual Focus of Attention* (VFOA). It represents who or what people are looking at. If two persons situated in the robot's FOV are looking at someone who is situated to the left/right of the robot (see Figure 4A), there is probably a reason and the robot should consider looking in that direction.

To calculate this stimulus, if two or more people in the robot's FOV are not looking at the robot and there is a difference between their pose vector below a given threshold, say 30°, the stimulus is increased for the people who are situated in the direction of the gaze of these persons.

#### 3.1.7. Stimulus *I*_*k*7_: Proxemics of a Person

Proxemics is the study of the human use of space. As stated by Hall et al. (1968), there are four distinct zones in the interpersonal relations: (1) intimate space, (2) personal space, (3) social space, and (4) public space. Following this proxemic approach, as in other works (Alonso-Mart́ın et al., 2012; Zaraki et al., 2014), the distance between the robot and a person is estimated. In each frame, this distance is proportional to the distance between the middle point of the eyes, points 37 and 46, and the end of the chin, point 9. A function that approximates this distance has been obtained, as follows:

(2) $$dp_{k}=-0.02\;\cdot \;\left|\vec{P_{m}(P_{37},P_{46}),P_{9}}\right|+2.04$$

where *dp*_*k*_ represents the distance between the robot and a person *k* in meters. Stimuli increased are those of people situated at the *far phase* of the personal space (0.76–1.22 m) or at the social space (1.22–3.70 m), as shown in Figure 4B.

This stimulus, *I*_*k*7_, is multiplied by an adjustment factor depending on the distance:

(3) $$f_{k}=\left\{ \begin{array}{l} 1\;if\;dp_{k}\text{>= 0.76 }\&dp_{k}\text{<= 2.10}\\ \frac{3.70-dp_{k}}{1.60}\;if\;dp_{k}>2.10\;dp_{k}<=3.70 \end{array}\right.$$

People situated at the *far phase* of the personal space (0.76–1.22 m) or at the *close phase* of the social space (1.22–2.10 m), multiply their stimulus by *f*_*k*_ = 1. People situated at the *far phase* of the social space (1.22– 3.70 m) multiply their stimulus by a factor, *f*_*k*_, which depends on the distance and is reduced until *dp*_*k*_ = 3.70*m*. These equations have been adjusted during experiments.

### 3.2. Competitive Network

When all stimuli have been obtained, a competitive network decides which person to gaze at. The competitive network creates a dynamic behavior between interlocutors. Instead of changing the gaze between participants abruptly, the network softens the change and avoids situations such as monopolizing attention. It has three components: an STM layer, which increases the duration of short stimuli; a Habituation layer, which penalizes persistent stimuli against the novel ones through a dynamic gain; and a Competitive layer, which creates a competition between participants and decides a winner.

**Figure 5:**
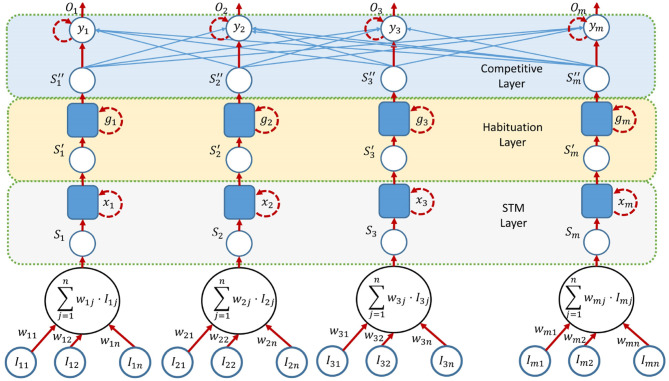
Architecture of the competitive network.

This network has one input and output for every possible person, as seen in Figure 5. Thus, *I*_*ij*_ corresponds to the stimulus *j* for the person *i* and *w*_*ij*_ to its associated weight. The weights take values between 0 and 1 and are chosen experimentally according to the relevance of the stimuli. The selected person is the one with the highest value in the output, *O*_*i*_. The network is composed of three interconnected layers.

One important aspect in this process is the configuration of the different parameters of such a neural network. The weights of the different stimuli have to be computed in order to achieve behavior similar to humans. During an initial training, three people follow a list of steps previously recorded. At the same time, another person observes the interaction and annotates the time instants when a person should be the focus of attention. When all data have been obtained, stimuli from expected winners and losers are separated based on the said manual annotation. The process is modeled as an optimization problem, maximizing the sum of the distances between the winners and the losers at each time instant *t*, as shown in Equation (4). This procedure ensures that the weights are optimal to make the selected persons winners and separate them from the losers.

(4) $$\begin{array}{l} \max\sum_{t=1}^{m}\left(\sum_{k\in losers}I_{t,winner}-I_{tk}\right)\\ =\max\sum_{t=1}^{m}\left[\sum_{k\in losers}\left(\begin{array}{l} \sum_{x=1}^{7}w_{x}.I_{t,winner},x\\ \;-\sum_{x=1}^{7}w_{x}.I_{t,k,x} \end{array}\right)\right] \end{array}$$

#### 3.2.1. Short Time Memory Layer

An STM increases the duration of the stimulus to have more value in the entry of the habituation layer. It is based on the model proposed by Grossberg (1982). The neuron activity is computed using Equation (5), where *x*_*i*_ is the activity of the neuron *i* and *A*_1_ is the decay rate. The next term is the auto-reinforcement, which makes neuron activity tend to its saturation value *B*_1_. *C*_1_ marks the growth rate. *S*_*i*_ is the filtered stimulus and *w*_*i*_ is the STM weight for that stimulus.

(5) $$\begin{array}{r} \frac{dx_{i}}{dt}=-A_{1}x_{i}+C_{1}\left(B_{1}-x_{i}\right)\left[S_{i}w_{i}\right] \end{array}$$

**Figure 6:**
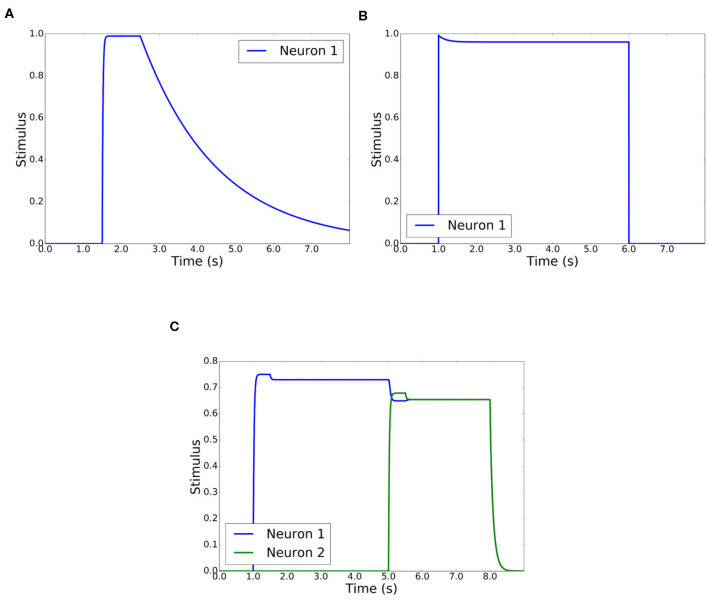
STM, habituation, and competitive layer behavior. **(A)** STM layer behavior when there is a short duration stimulus. *A*1 = 0.2, *B*1 = 1, and *C*1 = 0.5. **(B)** Habituation layer behavior when there is a long duration stimulus. *E* = 0.5, *F* = 0.02. **(C)** Behavior of competitive layer with 2 neurons. *A*2 = 1, *B*2 = 1, and *C*2 = 3, *D* = 1, *E* = 0.5, and *F* = 0.02.

Equation (5) is solved in real time using a trapezoidal integration defined by the following equations:

(6) $$\begin{array}{r} x_{i}\left(kh\right)=x_{i}\left(\left(k-1\right)h\right)+\frac{g_{i}\left(kh\right)+g_{i}\left(k-1\right)h}{2} \end{array}$$

(7) $$\begin{array}{r} g_{i}\left(kh\right)=-A_{1}x_{i}\left(kh\right)+C_{1}x_{i}\left(kh\right)\\ \;+C_{1}\left(B_{1}-x_{i}\left(kh\right)\right)\left[S_{i}w_{i}\right] \end{array}$$

where *k* represents an increasing value 0..*n* and *h* is the period of time (0.1 in experiments). Figure 6A reflects the behavior of the STM layer with parameters *A*_1_ = 0.2, *B*_1_ = 1, and *C*_1_ = 0.5. When a short stimulus arrives, the layer maintains its value during some time until it completely disappears.

#### 3.2.2. Habituation Layer

The proposed method has habituation capabilities, that is, it loses interest in permanent stimuli over time. Habituation networks were proposed by Grossberg (1968). In this layer, stimuli decay due to habituation allows the network to dynamically adapt against permanent inputs, such as a person who is hoarding the conversation. By using a habituation layer, those continuous stimuli lose preponderance over time, allowing novel stimuli to acquire more importance. Habituation is carried out by multiplying the input stimuli with a dynamic gain that is updated over time. The gain *g*_*i*_ computation is calculated based on Equation (8).

(8) $$\frac{dg_{i}}{dt}=E(1-g_{i})-FS_{i}^{\prime }g_{i}$$

In the same way as with the STM layer, the differential equation is integrated trough the following numerical discrete equations:

(9) $$g_{i}(kh)=g_{i}((k-1)h)+\frac{p(kh)+p((k-1)h))h}{2}$$

(10) $$p(kh)\;=E[1-g_{i}(kh)]-FS_{i}^{\prime }(kh)g_{i}(kh)$$

where $S_{i}^{\prime }$ is the filtered stimulus and *g*_*i*_ is the habituation gain for that stimulus. When a stimulus is active, the habituation gain decreases from the maximum value of 1 to a minimum value given by $E/\left(E+FS_{i}{}^{\prime }\right)$, proportional to the stimulus value $S_{i}^{\prime }$. This gain is recharged to its initial unity value when the stimulus ends. Charge and discharge rates are determined by the parameters *E* and *F*. Figure 6B shows the behavior of the Habituation layer with parameters *E* = 0.5 and *F* = 0.02. When there is a long stimulus in duration, the layer decreases its value to give more possibilities to other new stimuli from other people.

#### 3.2.3. Competitive Layer

The outputs of the habituation layer are the inputs of the competitive layer. The competitive model used is on-center off-surround (Grossberg, 1982) and is based on the model of Hodgkin (1952), where each neuron is reinforced with its own activity, but attenuated by the activity of the neurons it is connected to. This attenuation is known as lateral inhibition and makes people compete by means of the weight of their corresponding stimulus. Neuron activity is computed using Equations (11) and (12), where *y*_*i*_ is the activity of neuron *i* and *A*_2_ is the decay rate. The next term is the auto-reinforcement, which makes neuron activity tend to its saturation value *B*_2_. *C*_2_ marks the growth rate and $S_{i}^{\prime \prime }$ is the filtered stimulus. Finally, the last term represents lateral inhibition (off-surround).

(11) $$\begin{array}{l} \frac{dy_{i}}{dt}=-A_{2}y_{i}+C_{2}(B_{2}-y_{i})[S_{i}^{\prime \prime }+f(y_{i})]\\ \;-y_{i}\sum_{i\neq j}f(y_{j}) \end{array}$$

(12) $$\begin{array}{r} f\left(y_{i}\right)=D{y_{i}}^{2} \end{array}$$

As before, the differential equation is integrated trough the following numerical discrete equations:

(13) $$y_{i}(kh)=y_{i}((k-1)h)+\frac{q_{i}(kh)+q_{i}(k-1)h}{2}$$

(14) $$\begin{array}{l} q_{i}(kh)=\;=-A_{2}y_{i}(kh)+C_{2}y_{i}(kh)\\ \;+C_{2}(B_{2}-y_{i}(kh))[S_{i}^{\prime \prime }w_{i}]\\ \;-\sum_{i\neq j}Dy_{j}{\left((k-1)h\right)}^{2} \end{array}$$

A parabolic function has been selected for *f*(*y*_*i*_), so the winner neuron is reinforced against the rest. The competition schema is a winner-take-all, as it is desirable that only one person prevails over the complementary ones. Figure 6C shows the behavior of the competitive and habituation layers. The competitive layer has parameters *A*_2_ = 1, *B*_2_ = 1, *C*_2_ = 3, and *D* = 1, while the habituation layer has *E* = 0.5 and *F* = 0.02.

There are two inputs of stimuli. One input receives a stimulus of 0.7 that remains in time from *t* = 1 until *t* = 8. Another input receives the same stimulus, 0.7, from *t* = 5 until *t* = 8, where both stimuli disappear. The sequence of winners is: neuron 1 and neuron 2. Neuron 1 is the first winner, as it is the only stimulus. Because of the habituation layer, neuron 1 reduces its value and neuron 2 becomes winner, although the input stimuli are similar.

### 3.3. Angles of the Robot

An important step of the method is the transformation of the coordinates of a face into angles of the robot. These angles are situated in a universal coordinate system (UCS) and they remain in the robot's memory regardless of whether it turns its head. Kalman filters also use these angles to estimate the position and the robot accepts angles to move its head up/down and left/right.

Initially, the robotic head is situated in angles $\psi_{O}=0^{{}^{\circ}}$ and $\theta_{O}=0^{{}^{\circ}}$. When it turns its head, ψ_*c*_ and θ_*c*_ represent the center point of the image. This image is the robot's FoV. As seen before, DLIB returns a rectangle for each face detected in an image. The midpoint of that rectangle is (*x*_*k*_, *y*_*k*_) and corresponds to the person *k*.

**Figure 7:**
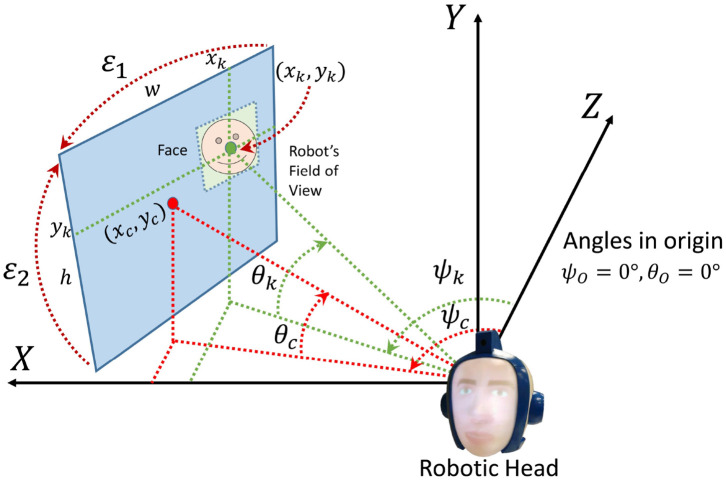
Transformation of the coordinates of a face into angles of the robot.

From (*x*_*k*_, *y*_*k*_), it is necessary to calculate the angles (ψ_*k*_, θ_*k*_). For that purpose, some fixed parameters are necessary. As seen in Figure 7, ε_1_ represents the vertical angle of the FoV, while ε_2_ corresponds to the horizontal one. *w* and *h* are the respective width and height of the image.

Equation (15) show the relation between (*x*_*k*_, *y*_*k*_) and (ψ_*k*_, θ_*k*_).

(15) $$\left\{ \begin{array}{l} \psi_{k}=\left[\psi_{c}-\frac{\varepsilon_{1}}{2}\right]-\left[x_{k}\cdot \frac{\varepsilon_{1}}{w}\right]\\ \theta_{k}=\left[\theta_{c}-\frac{\varepsilon_{2}}{2}\right]-\left[y_{k}\cdot \frac{\varepsilon_{2}}{h}\right] \end{array}\right.$$

Initially $\psi_{c}=\psi_{O}=0^{{}^{\circ}}$ and $\theta_{c}=\theta_{O}=0^{{}^{\circ}}$. When the robot turns its head, the target angles are aligned with the center of the image (*x*_*c*_, *y*_*c*_). Therefore, the robotic head moves to (ψ_*k*_, θ_*k*_) and, when it finishes, ψ_*c*_ takes the value ψ_*k*_ and θ_*c*_ takes the respective θ_*k*_. At that moment, (ψ_*c*_, θ_*c*_) are the current situation of the robot until the next movement.

### 3.4. Kalman Filter

The Kalman filter and the Extended Kalman filter (Rosales and Sclaroff, 1998) are well-known techniques that allow the position of a person to be estimated by means of positions known and previously updated. The Kalman filter considers noise and other inaccuracies and helps estimate the location. It uses Bayesian inference and estimates a joint probability distribution over the variables for each instant of time.

Several Kalman filters run simultaneously, one for each person previously tracked by the robot. When a new person appears, the proposed method considers an initial state vector with the first known positions of the person. This vector is composed of the corresponding angles of the robot calculated from the center point of the person's face, *x*_*k*_ = (ψ_*k*_, θ_*k*_). The Kalman filters are created with a posteriori error covariance matrix $P_{k}=\left|\begin{array}{cc} 1 & 0\\ 0 & 1 \end{array}\right|$(a measure of the estimated accuracy of the state estimate).

An iteration is produced when the person has been detected in a new frame and new positions/angles arrive. At that moment, the Kalman filter is updated with the new angles. *H*_*k*_ is the observation model which maps the true state space into the observed space. Specifically, the observation model is $H_{k}=\left|\begin{array}{cc} 0.1 & 0\\ 0 & 0.1 \end{array}\right|$ and the covariance of the observation noise is $R_{k}=\left|\begin{array}{cc} 0.25 & 0\\ 0 & 0.25 \end{array}\right|$

## 4. Robot Construction and Method Implementation

**Figure 8:**
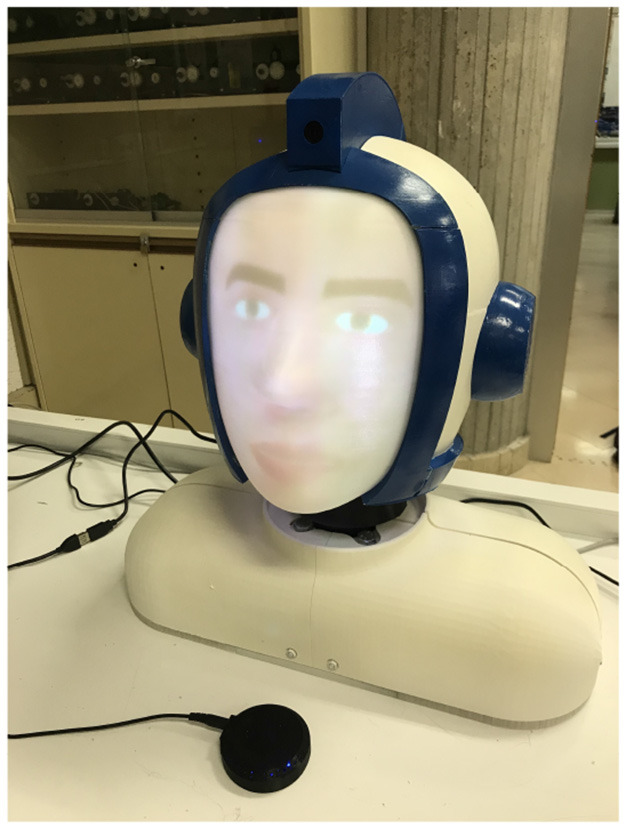
Robotic head developed.

A complete robotic head has been designed and built, as shown in Figure 8. The design considers the principle by which people will collaborate more naturally and easily with humanoid robots as compared with machine-like robots (Hinds et al., 2004), and the idea of the anticipated acceptance of a social robot when it provides more enjoyable interactions (de Graaf et al., 2019). This robotic head includes two servomotors for the orientation (ψ and θ angles) and a wide angle camera located at the top of the head. An ESP32 module has been programmed to move the servomotors, which are also connected to a step-down voltage regulator. The ESP32 module is connected to a computer, which is responsible for the robot's gaze control. The function developed in the ESP receives the target where the head has to be moved as ψ and θ angles. It moves the servos to that concrete position step by step and, each step, publishes the current position of the head.

A projector situated at the back projects an agent on a 3D printed display representing the robot's face. The agent follows an approach using the *Facial Action Coding System* (FACS) (Ekman, 1997), which is a well-known method for measuring and describing facial behavior. In FACS, several *Action Units* (AUs) are responsible for contracting groups of muscles in face changes.

**Figure 9:**
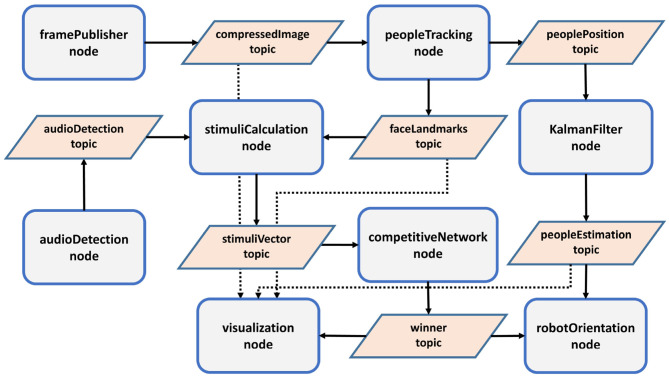
ROS architecture.

The computer in charge of processing the gaze control is an intel i9-9900K, with 32Gb of RAM and a GPU GeForce RTX 2080 Ti. It is connected to different elements: the ESP32 module, a mini projector with HDMI, and the circular microphone array, concretely a ReSpeaker Mic Array v2.0. This computer runs the *Robot Operating System* (ROS) (Quigley et al., 2009) over Linux, where the proposed method has been deployed. Several independent nodes run simultaneously and different messages are published and subscribed by these nodes. The architecture of the developed system is shown in Figure 9.

There are several nodes:

- *framePublisher*, which directly receives frames using OpenCV and publishes into a compressed image topic. It has a rate of 20 Hz. The image is transformed into a grayscale image and the dimensions are adjusted to an optimal value (500 pixels wide).
- *audioDetection*, which detects audio activity (AAD) and the direction (DOA). This node publishes a message, *audioDetection*, with a rate of 3 Hz, indicating both values. Other nodes with a different rate, such as *stimuliCalculation*, use the last received values, remaining in time until other, new ones are published. Our experimentation showed that this technique produced a better behavior in the recognition of a person speaking.
- *peopleTracking*, which is responsible for different activities:
  - Extracting people/faces/face landmarks from a frame using DLIB libraries.
  - Tracking people using face identification and a correlation tracker.
  - Obtaining people pose.
  - Keeping in memory a list of existing people, as well as their position, depending on the angles of the robotic head. The competitive network has 20 entries corresponding to possible interlocutors during the last activity of the robot. If the list is complete and a new person appears, the person not detected for the longest time is replaced by the new one.
  - Based on the list of existing people, publishing a message, *faceLandmarks* topic, with face characteristics (68 DLIB points, coordinates, pose) for the stimuli calculation and a message, *peoplePosition* topic, with new known positions for the Kalman filters.
- *stimuliCalculation*, which calculates the value of the entries of the competitive network using the information provided by the *faceLandmarks* topic. The stimulus *i* can take the values 0 or 1 for a person *k* and is multiplied by a weight *w*_*i*_ with the values represented in Table 1.

**Table 1:** Weights in stimuli calculations (obtained with the optimization problem).

**Stimulus *i***	***I*_*ki*_**	***w*_*i*_**
1. Person *k* detected in the robot's FOV	1/0	0.06
2. Person *k* speaking in the robot's FOV	1/0	0.25
3. Person *k* gazing directly at the robot	1/0	0.06
4. Person *k* moving sharply between frames	1/0	0.16
5. Person *k* not in the robot's FOV but with audio	1/0	0.25
6. Person *k* in the VFOA of other people	1/0	0.16
7. Proxemics of person *k*	1/0	0.06

All stimuli multiplied by their corresponding weights are accumulated for each person *k*, being normalized between 0 and 1, and they are published as a *stimuliVector* topic, which is the input of the competitive network.

This node performs another important task: when nobody is speaking in the robot's FOV, whenever there is audio in the left or right zone, the stimulus *I*_5_ is increased for a fictional person. There are two fictional persons who, respectively, have Kalman filters with angles in the left/right zone of the robotic head. This task is valid for situations where a new person appears to the left/right of the robot. As they have not been previously recognized by the robot, it does not take them into account. The use of the competition network includes them in the dynamic behavior process.

- *competitiveNetwork*, which implements the three previously explained layers:
  - *Short Time Memory layer (STM)*, which extends the duration of concrete stimuli.
  - *Habituation layer*, which penalizes persistent stimuli against novel ones.
  - *Competitive layer*, which decides the winner.

This node publishes a message with the winner, *winner* topic, which is in fact the stimuli vector processed by the network. The output with the highest value represents the winner.

- *Kalmanfilter*, which implements a Kalman filter for each different person. It receives the angles of the robot head for tracking a person, who is in the robot's FOV, and updates their corresponding values.
- *robotOrientation*, which is in charge of combining information received from the competitive network and the estimation of the Kalman filter, *peopleEstimation* topic, and sends the new angles to the robotic head. Another task is carried out by this node: when nobody has been detected during the last 20 s, the robot begins an exploratory movement whenever new audio is detected. The exploration initially moves the attention to the zone where the audio has been detected (left, central, right). During this exploration, the agent modifies the robot's expression from neutral to sad.

Based on the winner of the competition layer, the *robotOrientation* node obtains the target angles (ψ_*k*_ and θ_*k*_) of the winner *k*, from the Kalman filter. Before sending them to the ESP32 module, it makes some controls related to the agent movements:

- If the distance between (ψ_*k*_, θ_*k*_) and (ψ_*robot*_, θ_*robot*_), the current position of the robotic head, is lower than 10°, the node does not send the movement to the servos. Instead, a movement of the agent's eyes is produced in the direction of the target. This step avoids servo gittering.
- If that distance is over 10°, the node sends the movement to the servos and, at the same time, makes the agent smile and move the eyes in the direction of the target.

It is also interesting to mention that a blinking eyes movement has been given to the robot to show realism.

- *visualization*, which visually displays the results of the process on the computer screen.

## 5. Experiments and Results Discussion

An initial step consisted in determining the optimal parameters of the system. This step was carried out by means of the optimization problem previously described, where three people followed a list of established actions. An external observer followed the interaction and annotated the time instants when a person should be the focus of attention. The complete list of actions had 726 steps and the optimization problem was solved in 18 iterations and 0.23 s (in an intel i9-9900K) using the SLSQP algorithm (Kraft and Schnepper, 1989). The obtained results were *w*_1_ = 0.06, *w*_2_ = 0.25, *w*_3_ = 0.06, *w*_4_ = 0.16, *w*_5_ = 0.25, *w*_6_ = 0.16, and *w*_7_ = 0.06. It is important to mention that, depending on the behavior expected by the observer, some parameter may take slightly different values. After this step, several situations involving two/three interlocutors and the robotic head were considered. 13 interactions (with 31.200 frames) with two or three people were recorded, considering different stimuli. 8 people participated in the experiments repeating some of them with similar results. The results of 4 out of them are shown next.

**Figure 10:**
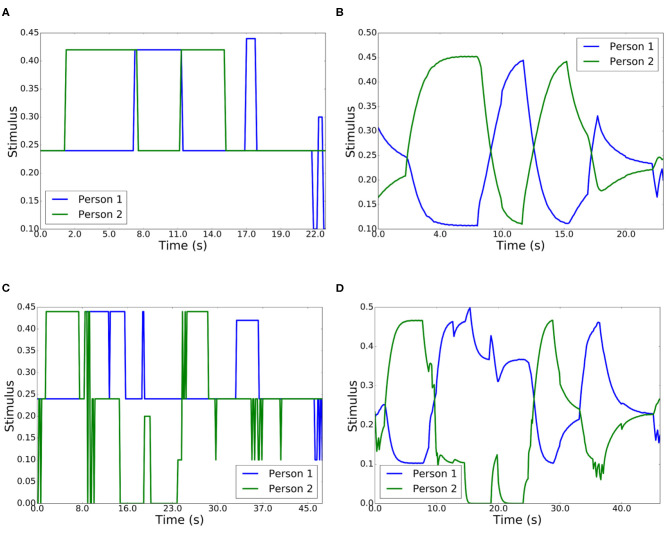
Robot interaction with two people. **(A)** Input stimuli in a conversation. **(B)** Output stimuli in a conversation. **(C)** Input stimuli with different actions. **(D)** Output stimuli with different actions.

During the first experiment, two people situated in the social space interacted with the robot. Figure 10A represents the summation of input stimuli in the competitive network, while Figure 10B shows the output. In this experiment, the two persons looked at the robot and had a conversation. Person 2 began the conversation (2–7 s), followed by 1 (6–12 s), 2 (12–15 s), and finally 1 (17–18 s). At the end of the conversation, the habituation capabilities appeared and person 2 became the winner (22–24 s). The output of the competition redirected the gaze of the robot dynamically.

During the second experiment with two people, a preset sequence of people's behavior was evaluated, as shown in Table 2. Depending on the stimuli obtained from both participants, the result of the competition was dynamically modified. This table shows the interval of time over which the stimulus takes place. The results of the winning person are shown in Figures 10C,D.

**Table 2:** Sequence of behavior of two people.

**State**	**Person speaking**		**Person gazing robot**		**Person moving**		**Winner**	**Time**
	**1**	**2**	**1**	**2**	**1**	**2**		
1		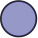	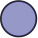	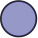			2	2–10 s
2	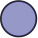		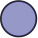	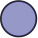			1	10–16 s
3			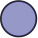				1	16–23 s
4		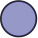	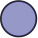	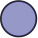			2	23–33 s
5			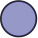	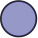	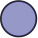		1	33–37 s
6				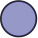			2	45–48 s

**Figure 11:**
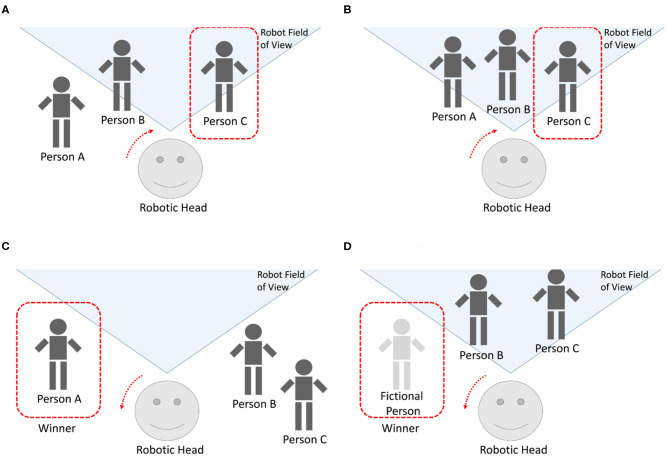
Different situations interacting with the robotic head. **(A)** Three people in the FOV. **(B)** Two people in the FOV. **(C)** Nobody in the FOV. **(D)** A new person appear on scene.

The following experiments were carried out with three people, as seen in Figure 11. In the case Figure 11A, there are two individuals in the robot's FOV. There is a conversation between three people on the scene and the robot moves its head depending on the winner, but, at a specific time, the stimuli produced by person C (speaking detection, gazing, proxemics, movement, etc.) and the result of the competitive network makes this person the winner and the robot turns its head to look at him/her. The second case, Figure 11B, is similar to the first situation but the three individuals are in the robot's FOV. The robot moves its head or eyes as soon as a new winner is elicited from the competitive network. As before, when person C becomes the winner, the robot turns its head right to center the person. The third case, Figure 11C, corresponds to a situation where the robot had previously detected three people. At a certain time, nobody is in the FOV, but the robot is detecting audio. The competitive network returns winner A and the robot turns its head left, according to the values returned by the Kalman filter associated to that person.

The fourth case, Figure 11D, represents two persons who are quiet in the robot's FOV. A new person arrives and begins to speak to the left. As soon as the robot detects sound in that zone, the fictional input of the competitive network corresponding to the left zone receives new stimulus. The competition evolves until that fictional person becomes the winner and, finally, the robot turns its head in that direction.

**Figure 12:**
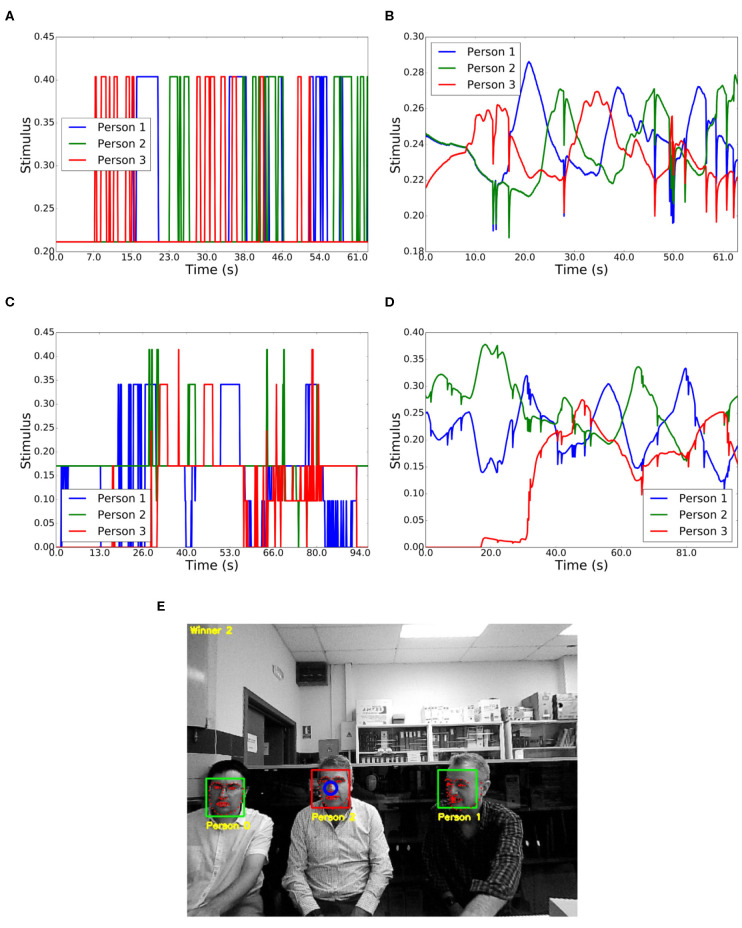
Interaction between three people. **(A)** Input stimuli in a conversation. **(B)** Output stimuli in a conversation. **(C)** Input stimuli with different actions. **(D)** Output stimuli with different actions. **(E)** Result of the *visualization* node showing the winner.

Figures 12A,B show the input and output values of the competition for the speaking interaction between three people. These three people were gazing at the robot and situated at the *close phase* of the social space (1.22–2.10 m). Person 3 began the conversation (10–17 s), followed by 1 (17–23 s), 2 (23–32 s), 3 (32–38s), 1 (38–42 s), 2 (42–48 s), 3 (48–53 s), 1 (53–60 s), and finally, 2 (60–63 s). During the seconds 35-38, person 3 was hoarding the conversation. However, person 1 began to speak and became the winner due to the habituation capabilities.

Table 3 shows, as in the case of two people, a preset sequence of people's behavior. Figures 12C,D show the corresponding input and output values for the competition, where several stimuli were considered, such as gazing at the robot, proxemics, speaking or moving. Figure 12E shows a moment of the experiment which is displayed by the *visualization* node.

**Table 3:** Sequence of behavior of three people.

**State**	**Detected/Proxemics**			**Gazing at robot**			**Moving**			**Speaking**			**Winner**	**Time**
	**1**	**2**	**3**	**1**	**2**	**3**	**1**	**2**	**3**	**1**	**2**	**3**		
1	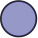	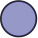			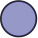								2	0–18 s
2	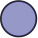	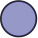	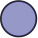	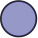	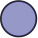								2	18–22 s
3	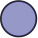	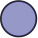	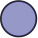	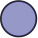	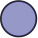		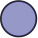						1	22–28 s
4	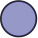	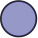	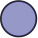	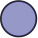	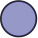			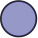			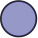		2	28–42 s
5	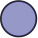	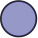	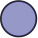	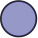	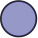	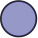			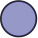				3	42–52 s
6	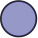	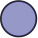	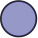	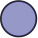	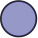	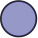	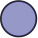						1	52–62 s
7	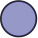	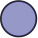	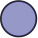		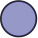						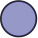		2	62–75 s
8	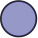	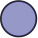	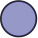	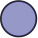								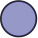	1	75–83 s
9	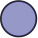	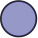	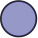		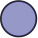	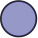							3	83–94 s
10	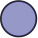	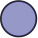		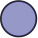	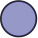								2	94–100 s

The proposed system reflects a successful result of 85.0% in relation to the behavior of a person in the same situation. To make this estimation, the result of the interaction has been supervised by comparing the selection of the focus of attention proposed by the system (winner nodes) and the one that would have been determined by a person observing the scene. The failures found were mainly due to errors in the perception of stimuli such as external audio noises unrelated to the interaction, and blurred images due to the effect of the movement of people and the robot itself. It is difficult to make a comparison of the results with other authors since there are no common datasets and each author uses different sensors and stimuli. A research work close to ours is that of Zaraki et al. (2014), where a precision between 75.2 and 89.4% is obtained depending on saccadic and non-saccadic movements. However, the conditions of the experiments are very different. In that work, a Kinect RGB-D and a DIK-ABLIS eye tracking system are used. In our experiments, a common webcam and an inexpensive ReSpeaker Mic array V2.0 are used. Moreover, in Zaraki et al. (2014) the interaction between 2 people at a certain distance is analyzed prioritizing gestural and postural acts, while our experimentation has been carried out at a short distance with the participation of three interlocutors (in fact, the number of interlocutors is not limited in the proposed method) and prioritizing facial and audio stimuli (where the people are looking, who is speaking, who is moving, their distance to the robot and even if someone gets out of the FOV).

Finally, mention that the program is available on the Internet at the URL:https://github.com/jaiduqdom/robotGazeControl.git.

## 6. Conclusions

This work presents a system to control the gaze of a robot interacting with multiple people in conversations. Several computer vision techniques have been used to obtain a set of stimuli, which are received by a competitive network that decides a winner and indicates where to look.

Different types of weighted stimuli have been considered, allowing the robot to focus its attention on one interlocutor. Thus, the proposed system identifies the interlocutors and re-identifies them when they leave the robot's FOV, tracks them using correlation tracking and Kalman filters, determines whether the person is speaking by identifying lip movement in concurrence with a sound source, studies where people are looking, monitors the person's movements to see if they wish to attract attention, and prioritizes close interlocutors over those further away. Kalman filters keep the angular positions of each particular person who has previously interacted with in the robot's memory, regardless of whether they are in the FOV or not.

The competitive network, by means of an adequate weighting of weights, allows who the robot should look at in order to create an interaction to be determined. The characteristics of the network allow a smooth transition between the focus of attention through the competition of stimuli. An important characteristic is the habituation mechanism that tends to prioritize new stimuli over existing ones and prevents a certain user from monopolizing the focus of attention.

Different experiments have been carried out in which two and three people have interacted with each other and with the robot. The results show how the competition of the network executed in real time allows a behavior of the robot similar to a person when choosing the focus of attention to be obtained.

A robotic head has been designed and built to evaluate the system, where a virtual agent projected on a 3D printed display has been used to represent the robot's face. Depending on the response of the gaze control system, the agent has shown different expressions and movement of the eyes. A ROS-based architecture has been presented and the different experiments carried out have been detailed.

The robot's behavior looks natural and is perceived similar to that of humans. The method has shown itself to be an important improvement in robot gaze control, creating a more realistic HRI system which is more acceptable to interlocutors than other robots that turn their heads without a dynamic and human-oriented method. The response of the competitive network has succeeded in producing soft transitions between different focuses of attention.

Finally, it can be noted that: people need to be properly detected by the face recognizer, which requires they are situated within a given distance range from the robot (2.2 m in our experiments); a GPUs is required for real-time processing (i.e., face recognition takes 0.4 s with CPU and 0.05 s with GPU in our experiments); and complexity of the statistical data analysis to perform inferential tests may differ from one person to another, due to inherent criteria differences.

The future objectives of the project will be the development of a conversational system, providing speech and voice recognition capabilities, and the perception and generation of emotions. These components, together with the developed gaze control system, will offer a low-cost, intelligent robot with human-like behavior. Simultaneously, the method will be available for other existing robots, since the ROS architecture means it can be integrated with other types of robots.

## Data Availability Statement

The program, a Video S1 and the experimental datasets (ROS badges) are available on the Internet at the URL: https://github.com/jaiduqdom/robotGazeControl.git.

## Ethics Statement

Written informed consent was obtained from the individual(s) for the publication of any potentially identifiable images or data included in this article.

## Author Contributions

JD-D, JG-G-B, and EZ conceived, designed and performed the experiments, analyzed the data, and wrote the paper.

### Conflict of Interest

The authors declare that the research was conducted in the absence of any commercial or financial relationships that could be construed as a potential conflict of interest.

## Supplementary Material

Video S1 Interaction with the robotic head.
